# Optimization and Technological Development Strategies of an Antimicrobial Extract from *Achyrocline alata* Assisted by Statistical Design

**DOI:** 10.1371/journal.pone.0118574

**Published:** 2015-02-24

**Authors:** Daniel P. Demarque, Sonia Maria F. Fitts, Amanda G. Boaretto, Júlio César Leite da Silva, Maria C. Vieira, Vanessa N. P. Franco, Caroline B. Teixeira, Mônica C. Toffoli-Kadri, Carlos A. Carollo

**Affiliations:** 1 Laboratório de Farmacognosia, Centro de Ciências Biológicas e da Saúde, Universidade Federal de Mato Grosso do Sul, Campo Grande, MS, Brazil; 2 Laboratório de Microbiologia, Centro de Ciências Biológicas e da Saúde, Universidade Federal de Mato Grosso do Sul, Campo Grande, MS, Brazil; 3 Faculdade de Odontologia, Universidade Federal de Mato Grosso do Sul, Campo Grande, MS, Brazil; 4 Faculdade de Ciências Agrárias, Universidade Federal da Grande Dourados, Dourados, MS, Brazil; 5 Laboratório de Farmacologia e Inflamação, Setor de Biofisiofarmacologia, Centro de Ciências Biológicas e da Saúde, Universidade Federal de Mato Grosso do Sul, Campo Grande, MS, Brazil; University of Toronto, CANADA

## Abstract

*Achyrocline alata*, known as Jateí-ka-há, is traditionally used to treat several health problems, including inflammations and infections. This study aimed to optimize an active extract against *Streptococcus mutans*, the main bacteria that causes caries. The extract was developed using an accelerated solvent extraction and chemometric calculations. Factorial design and response surface methodologies were used to determine the most important variables, such as active compound selectivity. The standardized extraction recovered 99% of the four main compounds, gnaphaliin, helipyrone, obtusifolin and lepidissipyrone, which represent 44% of the extract. The optimized extract of *A*. *alata* has a MIC of 62.5 μg/mL against *S*. *mutans* and could be used in mouth care products.

## Introduction

Natural products are an important source of bioactive compounds; however, many challenges exist when transforming them into phytotherapy medicines. The main difficulties involve identifying the bioactive compounds, the variations in secondary metabolites and the low reproducibility of traditional extractions [1]. The extractive method directly affects the pharmaceutical industry because it determines the quality and safety of the final product [2]. Modern techniques, such as accelerated solvent extraction (ASE) assisted by chemometric calculations are good alternate solutions to this problem and allow the extraction time, solvent quantity and yield to be optimized [3,4].

ASEs enable reproducible, exhaustive and stable extractions by using an inert nitrogen atmosphere with high temperature and pressure [5]. The parameter range allowed by the equipment yields extracts with different chemical and biological profiles. Furthermore, chemometric techniques, such as factorial experiments and a response surface method (RSM), can optimize the extractive variables and allow the monitored response variables to be understood, which yields the ideal extract for a specific purpose [6]. The monitored responses can be a chemical profile or biological activity as demonstrated by Zhou et al. [7], who used a DPPH (diphenyl-(2,4,6-trinitrophenyl) iminoazanium) antioxidant assay to establish the best production conditions of an antioxidant extracted from *Clerodendrum cyrtophyllum*.

Extract optimization must be based on the biologically active chemical constituents with aid of the ethnopharmacological use. A bioactivity assay could help identify the fraction/compounds responsible for an observed activity [8]; this step is required for widely used plants, such as species of the genus *Achyrocline* (Asteraceae), which are recommended for many diseases as inflammation and infections [9]. *Achyrocline alata*, a species of this genus found in Brazil, Paraguay and Argentina, contains various classes of secondary metabolites, such as flavonoids, terpenes and polyketides [10, 11]. Furthermore, the variety of traditional uses obfuscates which constituents must be present for each reported activity.

Hence, this study aimed to identify the antimicrobial metabolites in *A*. *alata* and standardize an active extract for use against *Streptococcus mutans*—the main bacteria that causes carie—using ASE and chemometric calculations.

## Materials and Methods

### Ethics Statement

The experiments were performed in accordance with the ethical principles for animal research adopted by the Ethics Committee on Animal Experimentation of the UFMS (CEUA/UFMS n°202/2009). Efforts were made to minimize suffering and after experimental procedures, the animals were euthanized in a CO_2_ chamber.

### Acquisition, drying and stabilization of plant material

*Achyrocline alata* (Kunth) DC was cultivated in the Universidade Federal da Grande Dourados, Dourados, MS, Brazil. A voucher specimen was deposited under registration number 40310 in the CGMS herbarium, Campo Grande, MS, Brazil. The drying process was performed at 40°C and the plant was powdered with a knife mill (20 mesh). Inflorescences of *A*. *satureioides* were bought from Chileno Chás e Ervas (Laboratório Industrial Vida e Saúde Ltda—Chapecó, Brazil) and were used without pretreatment.

### Hexane, ethanol, infusion and trichome extracts from *A*. *alata*

The hexane extract (HExt) was obtained from 0.6 g of the inflorescences using a model ASE-150 (Dionex) accelerated solvent extraction system with a 5 mL capacity cell using a hexane solvent at 130°C for a static extraction time of 4 minutes across 5 cycles with a 150% rinse. The ethanolic extract (EExt, 70% ethanol) was prepared using the same parameters sequentially from the same sample. The HExt was partitioned into a separatory funnel with hexane and 70% ethanol to obtain two phases: a hexane phase (HExt-HP) and ethanolic phase (HExt-EP). The trichome extract (TExt) was obtained using the method described by Schorr et al. [12]. The infusion extraction (IExt) involved pouring boiling water over the drug plant in a proportion of 2% for five minutes and then lyophilizing. All chemicals were analytical grade purchased from Vetec, Rio de Janeiro, Brazil.

### Ethanolic phase of *A*. *satureioides* and the steam/leaves extract from *A*. *alata*

The ethanolic phase extract from the *A*. *satureioides* inflorescences (HExt-EPsat) and steam/leaves extract (SLExt) from *A*. *alata* were obtained via an ASE using the final optimized method (95:5 hexane:ethyl acetate as extractor solvent, 150°C, one cycle, 1 minute static time and partition with 90% ethanol).

### Antibacterial activity against *Streptococcus mutans*

The antibacterial assay was conducted using HExt, EExt and HExt-EP. The *S*. *mutans* strain (UA159) was reactivated in a brain heart infusion broth (BHI, Sigma-Aldrich, São Paulo, Brazil) and the inoculum was standardized using a spectrophotometer at a wavelength of 625 nm to acquire a suspension containing approximately 1.5 × 10^6^ CFU/mL [13]. The extract samples were dissolved in 1% of dimethylsulfoxide (DMSO) (Vetec, Rio de Janeiro, Brazil) and diluted from 1000 μg/mL to 15.6 μg/mL by two-fold serial dilutions. The positive control used was 0.12% (v/v) chlorhexidine in water (Sigma-Aldrich, St. Louis, MO, USA) and 1% DMSO as the negative control. The microplates were incubated with BHI at 37°C in jars containing 5% CO_2_ for 24 hours.

### HPLC-DAD analysis

The HPLC-DAD analyses were performed using an SPD-M20A (Shimadzu, Kyoto, Japan) with a monolithic C-18 column (100 × 3 mm, Onyx/Phenomenex). Both water (A) and acetonitrile (B) (HPLC grade, Vetec, Rio de Janeiro, Brazil) containing 1% acetic acid were used as the mobile phases. This method began with 28% B for 5 minutes and increasing to 80% B over 11 minutes with a further 5.5 minutes to wash and stabilize the column at 1.8 mL/min flow.

### Analytical method validation

The method was validated in accordance with the International Conference on Harmonization of Technical Requirements for Registration of Pharmaceuticals for Human Use ICH-Q2B [14]. The specificity, linearity, range and accuracy were evaluated based on the gnaphaliin and helipyrone response. Standard curve had the correlation coefficient measured and specificity evaluated with the pick purity in HPLC and NMR ^1^H. Accuracy was determined by recovery of gnaphaliin and helipyrone added in triplicates on plant material previously cleaned, in concentrations of 10μg/ml, 25μg/ml and 50μg/ml, Reproducibility was determined by successive injections (6) in three different days and should not differ by more than 5%.

### Determination of Hydrogen peroxide and NO produced by peritoneal murine macrophages

The EExt and HExt-EP extracts were tested. Adult, male Swiss mice weighing 18–25 g were obtained from the animal colony in the Universidade Federal de Mato Grosso do Sul (UFMS), Campo Grande, MS, Brazil, maintained under controlled temperature (22 ± 2°C) and lighting (12/12 light/dark cycle), with water and food *ad libitum*. The six animals were kept in collective cages (40x 35x17 cm/ 3 animals/cage).

The macrophages were collected after pre-treating the mice with 3% thioglycolate 96 hours before harvesting. These cells were obtained by washing the cavities and a total cell count was obtained using a Neubauer hemocytometer. The cells were resuspended in an RPMI 1640 medium (Sigma Aldrich, St. Louis, USA) supplemented with 100 U/mL of penicillin, 100 μg/mL of streptomycin and 10% fetal bovine serum (Sigma-Aldrich, St. Louis, USA).

The macrophage suspension (2×10^5^ cells/100 μL) was added to the bottom of 96 wells and incubated for 1 hour at 37°C in a CO_2_ incubator for cell adhesion. Subsequently, the non-adherent cells were removed, and 100 μL of a phenol red solution (140 mM NaCl, 10 mM potassium-phosphate buffer pH 7.0, 5.5 mM dextrose, 0.56 mM phenol red) containing 8.5 U/ml of horseradish peroxidase (Sigma Aldrich, St. Louis, USA) and the EExt and HExt-EP in the presence or absence of phorbol myristate acetate (PMA, 50 ng/well, Sigma Aldrich, St. Louis, USA, 10 μL) was added, and the cells were incubated for 60 minutes at 37°C in a 5% CO_2_ atmosphere. The absorbance was determined using an ELISA reader at a wavelength of 620 nm [15].

The technique used to determine the release of nitric oxide was described by Ding et al. [16]. The non-adherent cells were removed and LPS (1μg/mL) and/or the EExt and HExt-EP were diluted in supplemented RPMI 1640 medium and added to the plate. The cells were incubated for 48 h, and at the end of this period, the production of NO was determined by the accumulation of nitrite in the cell culture supernatants. The absorbance was determined using an ELISA reader at a wavelength of 540 nm. These assays were performed in triplicate while simultaneously maintaining control cells without any PMA and lipopolysaccharide (LPS) for analysis of the spontaneous release of hydrogen peroxide and nitric oxide, respectively.

The cell viability was performed by mitochondrial-dependent reduction of MTT [3-(4,5-dimethylthiazol-2-yl)-2,5-diphenyltetrazolium bromide] (Sigma-Aldrich, St. Louis, USA) to formazan [17]. The peritoneal macrophages were obtained as previously described, incubated with the same conditions for 24 hours. Then, cells were incubated with EExt (4mg/mL, 8 mg/mL, 0.16 mg/mL) and with HExt-EP (4 mg/mL, 0.8 mg/mL, 0.16 mg/mL) for 16h. After this period the MTT (20μL, 5mg/mL) was added, incubed for 2 hours and absorbance was determined using an ELISA reader at a wavelength of 540 nm.

### Variables screening by factorial design

The factorial design was built in a 2^5^ factorial model, resolution III and in duplicates to optimize the follow variables: extractor solvent (hexane/acetone), temperature (70 to 130°C), cycles (1 to 5), time (1 to 5 min) and ethanol/water partition (50–90%). The response monitoring was based on the HExt-EP extract yields for active and inactive compounds (S1 Table).

### Temperature and solvent optimization via RSM experimental design

The temperature (100°C to 180°C) and extractor solvent (hexane:ethyl acetate 50:50–100:0) were optimized via the RSM experimental design using a larger amplitude and a different solvent polarity to a fine-tuning of these parameters. The others parameters previously optimized via the factorial design were kept. These response analyses were based on the gnaphaliin and helipyrone concentration—quantified via HPLC—and extract yield (S2 Table).

### Scale-up and exhaustive extraction of active chemicals

The exhaustive extraction and scale-up analyses were performed sequentially three times using 0.6 g of plant in a 5 mL cell and 5 g in a 100 mL cell according to the standard method. A comparison was made using the gnaphaliin and helipyrone concentration.

### Isolation of HExt-EP compounds by semi-preparative HPLC-DAD and analysis by NMR and ESI-MS

The HExt-EP (600 mg) was fractionated in an HPLC-DAD Shimadzu, model LC-6AD, with a C-18 column (250 mm x 20 mm, Shimadzu), water (A) and acetonitrile (B), both with 0.05% TFA as mobile phase. The methodology started with 70% B for 6 minutes increasing to 95% B until 26 minutes and 7 minutes to stabilization of column, at 10.5 mL/min flow rate. The peaks with retention time (RT) 9.8 min; 15.7 min; 20 min and 22.23 min were manually collected.

The compounds were identified by ^1^H, ^13^C and DEPT-135° NMR, Bruker DPX-300 (300/75 MHz) diluted in deuterated chloroform or deuterated methanol (Merck, Darmstadt, Germany) and mass spectrometry, high resolution (microTOF II-ESI-TOF—Bruker Daltonics) with positive and negative ionization mode.

### DPPH antioxidant assay

The antioxidant assay was made in 96-well plate described by Herald et al. [18]. EExt, HExt-EP and quercetin (400 μg/mL) as positive control were tested. The aqueous solution of DPPH (0.1 mMol) was prepared and 50 μL was added to all wells except the solvent wells, in which 50 μL of methanol was added. In the blank wells were added 25 μL of methanol and 50 μL of DPPH. In the sample wells were added 25 μL of the sample and 50 μL of DPPH in at least ten dilutions. Absorbance was measured at 517 nm using a microplate reader, SpectraMax Plus^384^, Molecular Devices, after 1.5 hours. The percentage of DPPH quenched was determined according to the equation: [(A_sample_-A_blank_)x100/A_DPPH blank_].

### Statistics

The factorial design, RSM design and Probit analysis of antioxidant test were performed using Minitab 16, Minitab Software Inc., USA. Statistical analyzes evaluating the production of reactive oxygen and nitrogen species by murine macrophages were analyzed by ANOVA followed by Bonferroni test using GraphPad Prism 5, GraphPad Software Inc., USA. The P values less than 0.05 were considered statistically significant.

## Results

### Extracts, fraction yields and microbiological assays

The EExt and HExt yields were 9.89 ± 3.5% and 4.35 ± 0.3%, respectively. After partitioning HExt, the HExt-EP and HExt-HP had yields of 1.8 ± 0.5% and 1.34 ± 0.5%, respectively. The IExt, TExt, HExt-HPsat and SLExt yielded 8.8%, 5.14 ± 1.1%, 2.1 ± 0.4% and 0.8%, respectively. All the results were based on the dry drug vegetable.

HExt exhibited antimicrobial activity with an MIC of 500 μg/mL, while the EExt extract exhibited an MIC of 1000 μg/mL. Thus, constituents present in the HExt were investigated to establish the chemical profile responsible for this antimicrobial activity. The HExt was partitioned using hexane and ethanol, and the HExt-HP and HExt-EP phases were obtained. Only the HExt-EP extract could be tested because HExt-HP was insoluble. HExt-EP had an MIC of 62.5 μg/mL (S3 Table).

### Isolation and identification of the HExt-EP compounds

**Fig 1:**
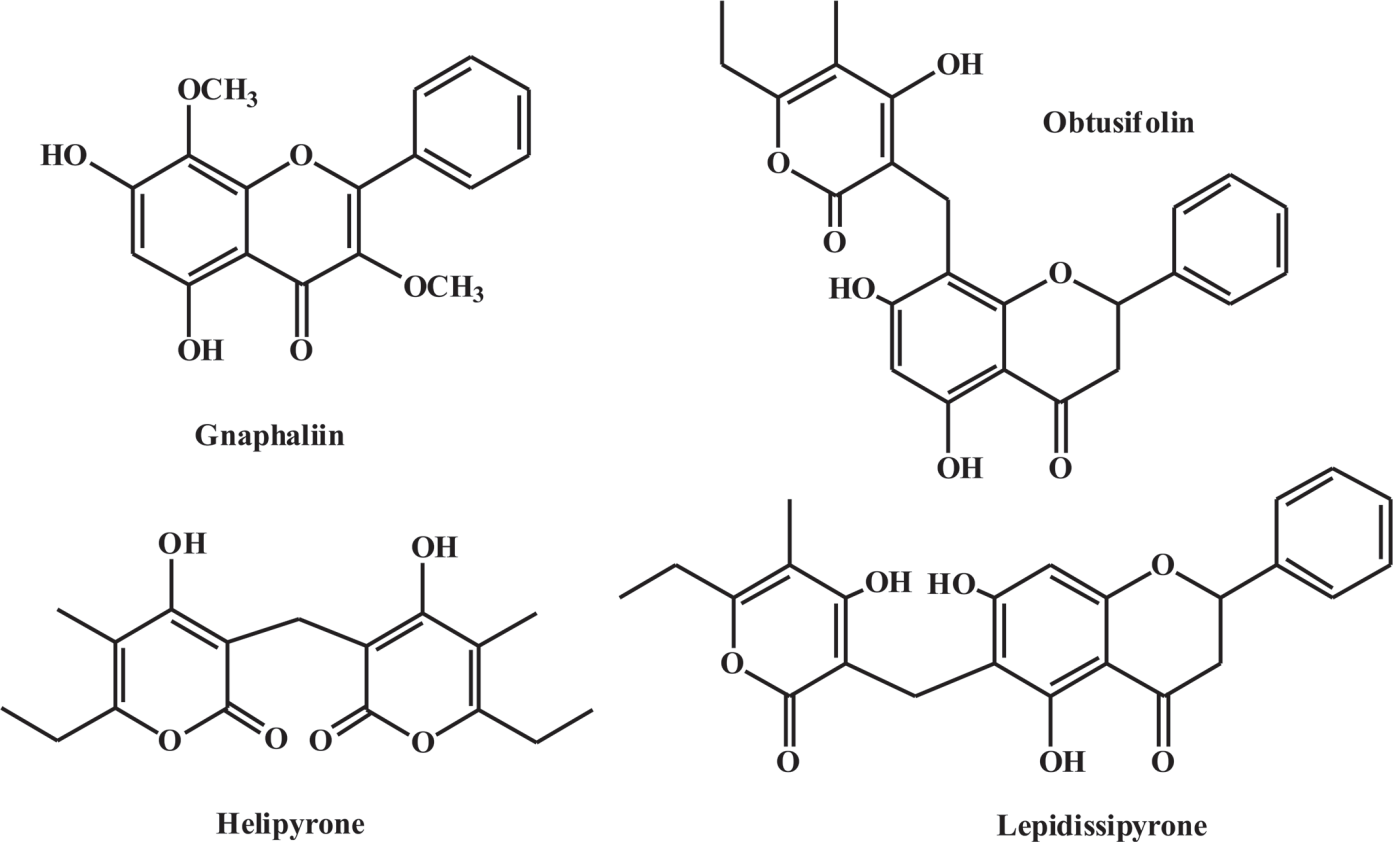
Chemical structures of the main identified compounds.

The compound with a retention time (RT) of 9.69 min was identified as the flavonol gnaphaliin (99 mg). The compounds with RTs of 10.1 min and 11.3 min were identified as the flavanones lepidissipyrone (30.9 mg) and obtusifolin (21.9 mg), respectively. The structural difference between these molecules is the position of the pyrone group—in lepidissipyrone this group is linked at C-6, while this group is attached to C-8 in obtusifolin. The compound with an RT of 10.5 min was identified as the polyketide helipyrone (65.2 mg), which contains the same pyrone unit as the flavanones (Fig. 1) (NMR data see S1 File).

### Extract standardization and analytical method validation

A standardized *A*. *alata* extract for use against *S*. *mutans* must contain at least 14 ± 5% of gnaphaliin and at least 20 ± 4% of helipyrone. This profile was established based on the HExt-EP analyses (the fraction with the best activity against *S*. *mutans*). The specificity was confirmed via UV peak purity calculations for gnaphaliin and helipyrone in the extract and isolate. The corresponding linear regression founded had an R of 0.9998 for both of the isolated compounds. The range maintained a coefficient of variation below 5% and the accuracy was acceptable with coefficients of variation below 5%.

### Variables screening by factorial design

**Fig 2:**
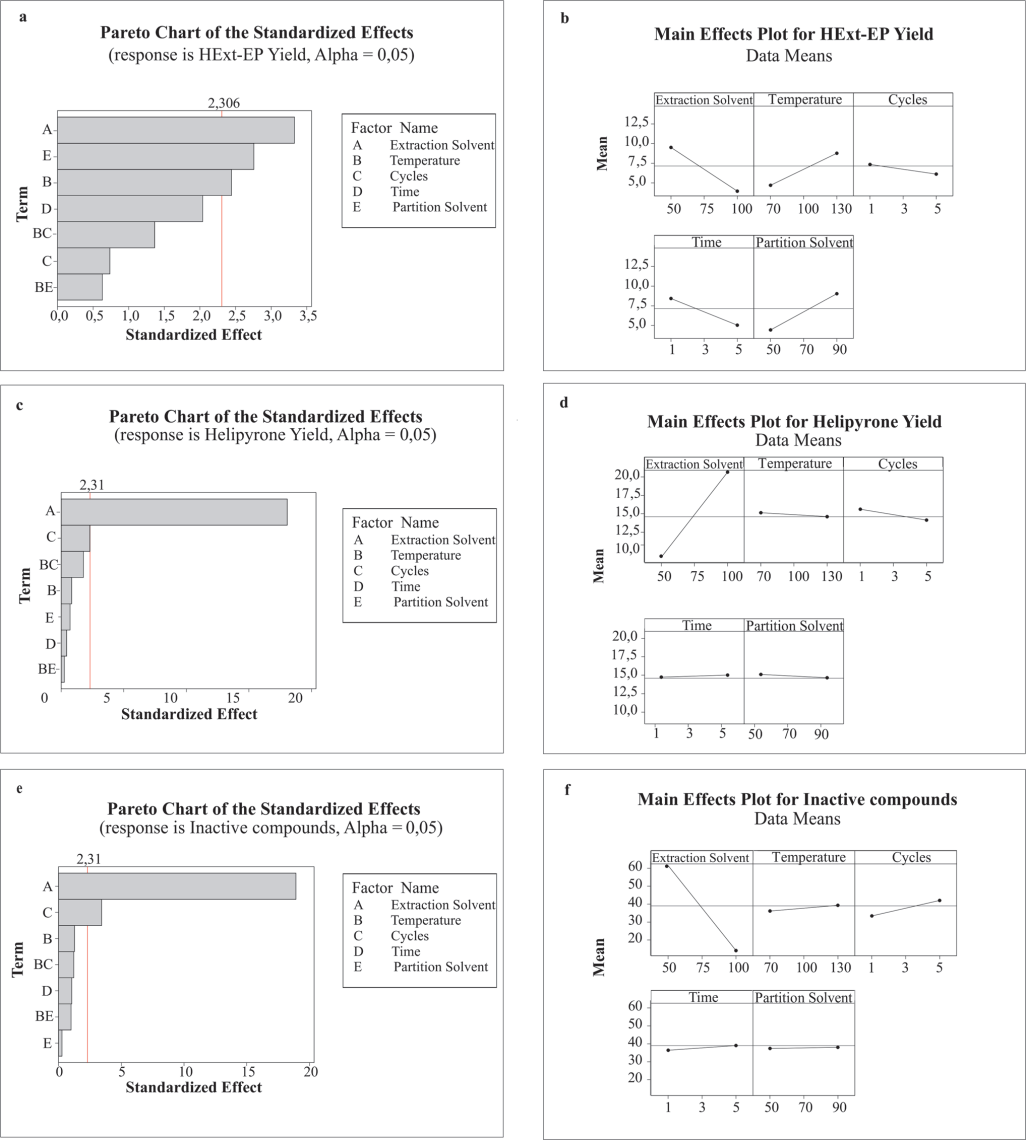
Pareto Chart of the standardized effects and main effects plot for HExt-EP yield (A and B), helipyrone yield (C and D) and inactive compound yield (E and F).

Of the five analyzed factors, the following were significant to the extract yield: the extractor solvent, temperature and ethanol percentage during partitioning, as shown on the Pareto chart of standardized effects (Fig. 2A). The effects plot figure (Fig. 2B) shows a negative influence of hexane (−5.55) on the extract yield. Increasing the temperature and ethanol percentage in the partition also positively contributes to the yield.

The Pareto chart of standardized effects in response to the active compounds indicated the solvent is the only significant parameter (Fig. 2C). However, this effect is opposite that found for the HExt-EP yield (Fig. 2D). Consequently, HExt-EP yield is was favored by acetone in the extractor solvent, while helipyrone and other active compounds yield require more hexane.

The third analyzed response was the inactive metabolites. In the Pareto chart of standardized effects (Fig. 2E), the most significant factors were the extractor solvent and cycle. Fig. 2F shows that the extractor solvent had a negative effect (−47.32), which proves increasing the percent acetone and cycles increases the inactive product yield.

The best parameters for increasing the yield and selectivity for the compounds of interest are 100% hexane as the extractor solvent, 130°C, one cycle and 90% ethanol partition. The time was not a statistically significant parameter.

### Temperature and solvent optimization via the RSM experimental design

**Fig 3:**
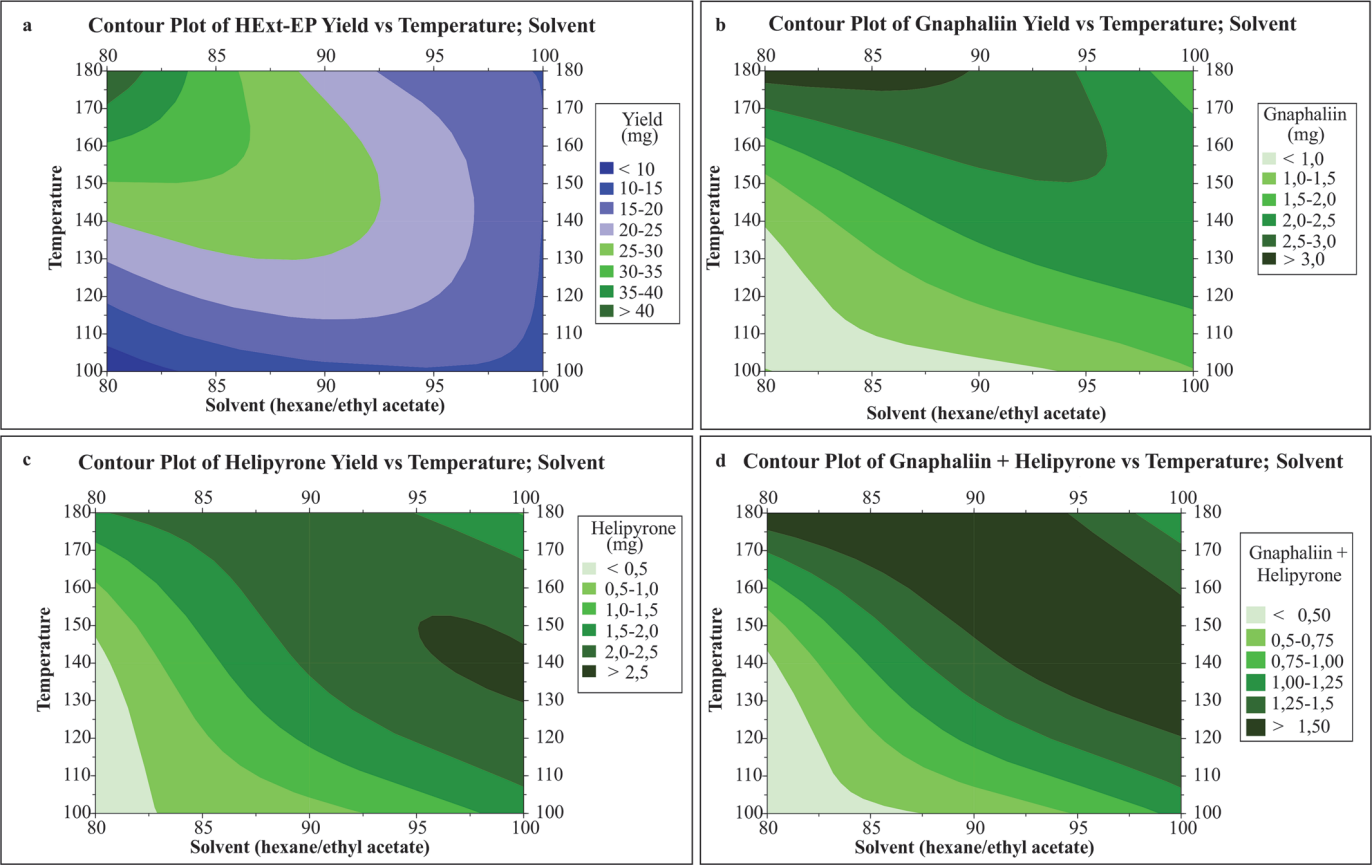
Contour plot of responses analysied on during the RSM experiment for HExt-EP yield (A), Gnaphaliin yield (B), Helipyrone yield (C) and the sum of gnaphaliin and helipyrone (D).

While optimizing the temperature and solvent via the RSM, the solvent extractor was changed from acetone to ethyl acetate because of its lower relative polarity and due to a sealing ring in the ASE extractor cell that does not support acetone at temperatures above 130°C. The maximum percentages of compounds were calculated from these results both alone and together (Fig. 3).

The HExt-EP (Fig. 3A) had the best yield when the temperature and solvent were 180°C and 80% hexane, respectively. This result was expected based on the responses previously observed during the factorial design, where higher temperatures and more polar extractor solvents increased the final extract yield.

The Fig. 3B shows the gnaphaliin yield, which resembling more to HExt-EP optimum yield. The combination of high temperatures and polar solvents tends to extract more polar compounds. Fig. 3C shows the ideal helipyrone region was between 95–100% hexane with a temperatures range from 130 to 150°C.

Fig. 3D shows the total gnaphaliin and helipyrone yield, and the darker region indicates where the maximum yields of both compounds can be obtained. Therefore, the final method for maximizing the yield and selectivity of the active compounds was 95% hexane as the extractor solvent, 150°C, one cycle, 1 minute of static time and at 90% ethanol partition.

### Scale-up and exhaustive extraction of active chemicals

The present work established the ideal production conditions for a standardized extract with a maximum yield, selectivity and activity against *S*. *mutans*. The exhaustive extraction and scale-up analysis proves the optimal parameters adopted by the final methodology founded on the chemometric analysis. The first extraction yielded 98.83% of gnaphaliin and 99.05% of helipyrone. The scale-up, changing the cell by 100 mL cell, provided on the first extraction 94% of yield for both substances monitored (Table 1).

**Table 1:** Gnaphaliin and Helipyrone yield in successive extractions with the optimized method and in scale-up.

**5ml Cell (0.6g)**	**Gnaphaliin yield (%)**	**Helipyrone yield (%)**
**Extraction 1**	98.83	99.05
**Extraction 2**	0.96	0.72
**Extraction 3**	0.19	0.22
**100ml Cell (5g)**		
**Extraction 1**	94.29	94.18
**Extraction 2**	2.74	2.85
**Extraction 3**	2.95	2.96

### Hydrogen peroxide and NO production by peritoneal murine macrophages

The EExt did not change the H_2_O_2_ produced by the macrophages or PMA and potentiated NO liberation due to LPS stimulation at 0.16 mg/mL (Table 2). The HExt-EP extract potentiates H_2_O_2_ liberation at 0.16 mg/mL but only in the presence of PMA. The NO liberation concentrations of 0.16 mg/mL and 0.8 mg/mL indicate the effect of LPS on the stimulated macrophage. The optimized extract HExt-EP was not cytotoxic to the cells during the MTT (3-(4,5-Dimethylthiazol-2-yl)-2,5-Diphenyltetrazolium Bromide) assay, where EExt 4mg/mL shows 19% of viable; 0.8 mg/mL (44%); 0.16 mg/mL (91%) and for HExt-EP 4 mg/mL (90%); 0.8 mg/mL (91%); 0.16 mg/mL (90%).

**Table 2:** Effect of the EExt and HExt-EP extracts on hydrogen peroxide (H_2_O_2_) production and nitric oxide (NO) production by mouse peritoneal macrophages.

Treatment	μM H_2_O_2_	Treatment	μM NO
Macrophages (MF) MF + PMA 50ng	**0.6 ± 0.37 30.10 ± 6.23** *	**Macrophages (MF) MF + LPS 1**μ**g/mL**	5.27±0.01 16.79±0.04**
**Eext**			
MF + 0.16mg/mL	**0.51 ± 0.51**	**MF + 0.16mg/mL**	5.92± 3.03
MF + 0.8mg/mL	**0.51 ± 0.51**	**MF + 0.8mg/mL**	3.85± 0.76
MF + 4mg/mL	**10.65 ± 2.30**	**MF + 4mg/mL**	4.40±0.98
MF + PMA + 0.16mg/mL	**21.60 ± 5.73**	**MF + LPS + 0.16mg/mL**	24.67±3.56**
MF + PMA + 0.8mg/mL	**24.60 ± 3.95**	**MF + LPS + 0.8mg/mL**	7.58±3.62
MF + PMA + 4mg/mL	**39.65 ± 6.65**	**MF + LPS + 4mg/mL**	5.70±1.63
**HExt-EP**			
MF + 0.16mg/mL	**4.81 ± 1.64**	**MF + 0.16mg/mL**	14.69±8.13
MF + 0.8mg/mL	**3.41 ± 1.92**	**MF + 0.8mg/mL**	9.93±5.75
MF + 4mg/mL	**0 ± 0**	**MF + 4mg/mL**	6.37±1.11
MF + PMA + 0.16mg/mL	**62.46 ± 1.94** *	**MF + LPS + 0.16mg/mL**	39.18±0.76**
MF + PMA + 0,8mg/mL	**38.20 ± 3.82**	**MF + LPS + 0.8mg/mL**	43.52±0.11**
MF + PMA + 4mg/mL	30.70 ± 4.16	MF + LPS + 4mg/mL	24.12±3.25

n = 3 performed in triplicate;

* P<0.05 compared to macrophages (MF).

** P<0.05 compared to macrophages estimulated with LPS. ANOVA and Bonferroni’s test. PMA (phorbol myristate acetate); lipopolysaccharide (LPS); Macrophages (MF).

### Antioxidant assay

The DPPH assay indicates that EExt (IC_50_ = 19.8) and quercetin (IC_50_ = 3.4) shows good antioxidant activity, while the standardized HExt-EP did not has the same potential (IC_50_ = 609) (S3 Table).

### Chemical profiles of HExt-EP, STExt, TExt and IExt extracts from *A*. *alata* and HExt-EP*sat* from *A*. *satureioides*

HExt-EP, TExt and STExt had similar chemical profiles. The 4 compounds identified in this study are also present in IExt, although they only consist of 4% of the extract. *A*. *satureioides* shows a different and more complex chemical profile than *A*. *alata*.

## Discussion

The results demonstrated the efficacy of accelerated solvent extraction with the aid of chemometric calculations and bioactivity assays for obtaining an *A*. *alata* optimized extract with potent antimicrobial activity.

Chlorhexidine exhibits a MIC of 2.7 to 80 μg/mL [19] for different types of bacteria and for *S*. *mutans* (UA159) was founded a MIC of 1.25 μg/mL [20]. Other compounds used in oral products like triclosan has a MIC ranging 0.1–20 μg/mL for some strains of *S*. *mutans* [21] and HExt-EP had a MIC of 62.5 μg/mL. Palombo [21] stated that vegetable extracts with MIC above 100 μg/mL has potential to develop products with oral applications. And the present results demonstrates the potential usefulness of HExt-EP against *S*. *mutans*. Other bacteria must be tested for new applications of the optimized extract.

The four main components in HExt-EP constituted approximately 44% of the extract. The flavonoid gnaphaliin was characterized as being antifungal against *Botrytis cinerea* [22], reduced both edema and leukocytes infiltration [23], which modulated the anti-inflammatory activity, and inhibited human low-density lipoproteins [24]. In present work, three new compounds were characterized from this genus: the flavanones obtusifolin and lepidissipyrone and the polyketide helipyrone. Obtusifolin was first described by Hänsel et al. [25] while studying *Gnaphalium obtusifolium* (Asteraceae), and lepidissipyrone was identified in the genus *Helichrysum* (Asteraceae) [26]. Helipyrone was first found in *Helichrysum italicum* [27].

Flavonoid prenylations or substitutions at the C-6 and C-8 positions may increase the antimicrobial activity [28] by rendering the molecule less polar and enhancing the bacterial penetration. Tsuchiya and Iinuma [29] found the antimicrobial mechanism for this molecule type/class could be involved in reducing the membrane fluidity of gram-positive bacteria.

Helipyrone polyketide exhibited an MIC of 6 μg/mL against *Bacillus subtilis*, *Staphylococcus aureus*, *S*. *epidermidis* (gram-positives) and the yeast *Mycobacterium phlei* and an MIC of 100 μg/mL for the gram-negative bacterias *Escherichia coli*, *Klebsiella pneumoniae*, *Pseudomonas aeruginosa* and *Salmonella typhimurium* [30]. The selectivity for gram-positive bacteria supports the importance of helipyrone to HExt-EP and its monitoring when optimizing the extraction for use against *S*. *mutans*. Studies utilizing bioactivity assays as a tool for standardization are successful because they can find better parameters to improve the yield of interesting compounds. Herrero et al [31] show that this method is not only applicable to plants. These authors use a factorial design and accelerated solvent extraction to monitor antioxidants compounds extracted from the microalga *Spirulina platensis*.

The hydrogen peroxide/NO production by peritoneal murine macrophages shows an atypical result for HExt-EP, where at higher concentrations induces a decreasing response (Table 2). The literature has reported several compounds that induce a biphasic response, like the agonist agent PMA [32], LPS [33,34], garlic extract [35], IL-1 [36] and others [37]; and in all these researches, increasing the dose of stimulators a decreasing effect is observed. Therefore, HExt-EP may contain compounds that induce this response. In addition, the extracts concentration ranged, keeping the stimulus concentration uniform and this must contribute to biphasic response. When this occurs may, perhaps, exist a synergic effect between the via involved with counterregulation response and the system protects itself against cytotoxic agents.

The antibacterial activity of HExt-EP can be better understood using the results from the hydrogen peroxide/NO production of peritoneal murine macrophages and antioxidant assays. Increasing the reactive oxygen species added with lower antioxidant activity suggests that HExt-EP may act *in vivo* directly as a bacteriostatic and indirectly by stimulating the immunologic responses of the macrophages [38, 39]. These results are complementary considering the necessity of immune responses for eliminating pathogens via bacteriostatic agents [40].

The standardized HExt-EP was more effective for eliminating *S*. *mutans* than the IExt, which had 4% of the identified compounds versus 44% for HExt-EP. Moreover, the compounds identified in this study represent 0.4% of the dry plant weight in IExt, whereas they constitute 0.9% of the dry plant weight in HExt-EP. In addition to the improved selectivity, the HExt-EP extracted twice as many compounds of the infuse extraction, reduced the antioxidant activity and stimulated the macrophages to produce reactive oxygen species, which are important to the immune response against bacteria [41].

Comparing the results with Toffoli-Kadri et al. [42] evidences the importance of selective extraction for a specific activity apparent. The hydromethanolic extract from *A*. *alata*, which has a similar chemical profile to EExt (data not shown), inhibited the reactive oxygen species and has potent antioxidant activity [43]. Thus, the standardizing the extracts for biological purposes may ensure their therapeutic success considering using different extraction methods on the same drug vegetable may generate distinct chemical profiles and even opposite biological responses.

**Fig 4:**
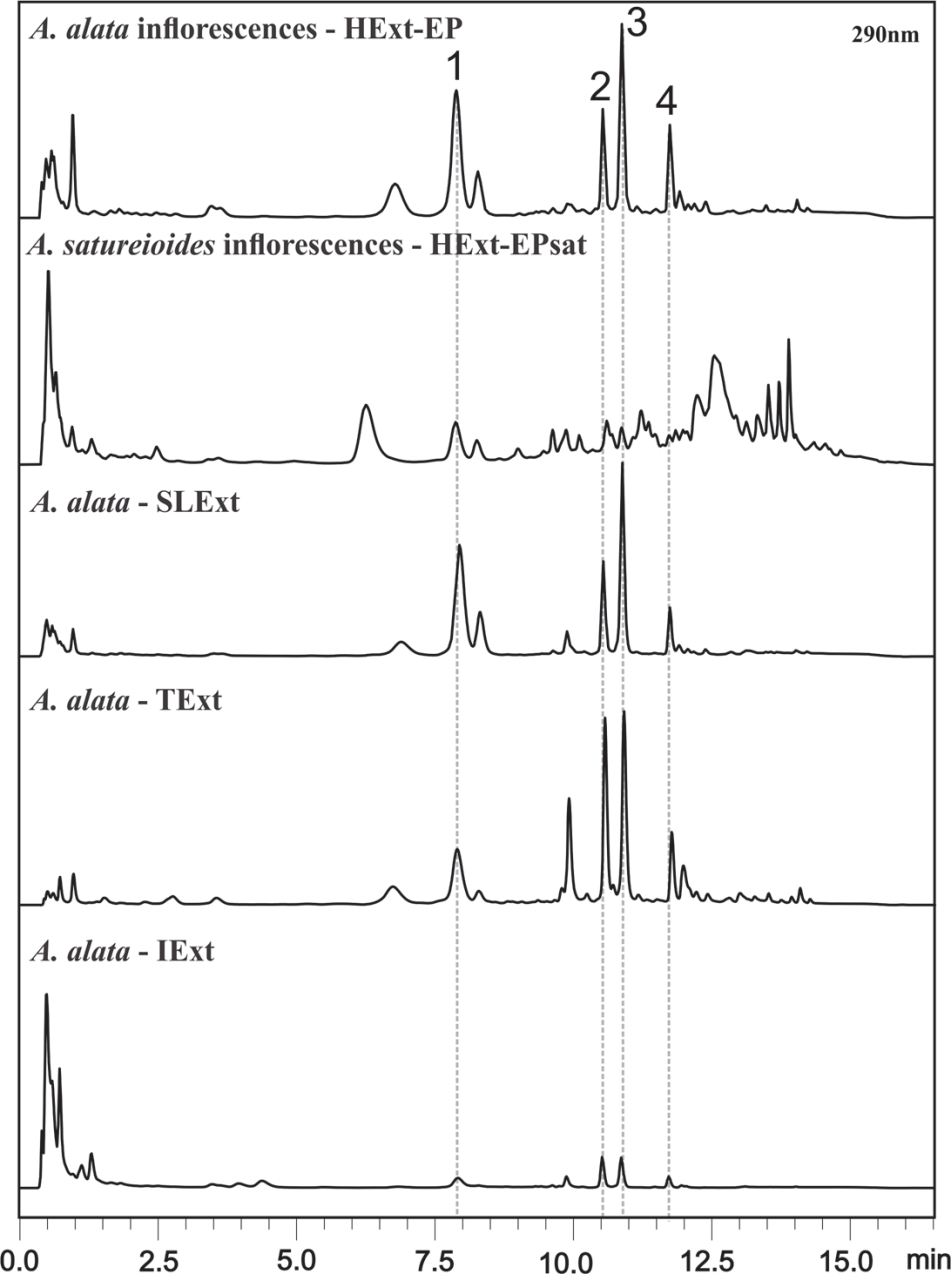
Chromatograms HPLC-DAD of HExt-EP, HExt-EPsat, SLExt, TExt and HExt at 290 nm. The compounds Gnaphaliin, Lepidissipyrone, Helipyrone e Obtusifolin is indicated by the numbers 1, 2, 3 and 4, respectively.

A comparison of HExt-EP from *A*. *alata* and HExt-EPsat from *A*. *satureioides* shows distinct chemical profiles. The infusion of *A*. *alata* inflorescences has been used as substitute of *A*. *satureioides* to treat infections and inflammations and *A*. *satureioides* was used to evaluate the interchangeable of the species for the optimized extraction method. *A*. *satureioides* has a complex chemical profile, while *A*. *alata* shows only four major compounds (Fig. 4), which differ in similarity between their polar compounds [10]. This extraction can be used to effectively identify these species.

The MTT assay is essential for evaluating the cellular response against different treatment parameters because cell viability below 80% is able to effectively respond to a specific stimulus and leads to differentiation in inflammatory macrophages [44]. The optimized extract HExt-EP proves the cell viability of HExt-EP at all concentrations. The use ASE extractor coupled with chemometric calculations was effectively to optimized yield, selectivity, antimicrobial activity and allows obtaining a safety extract compared to the EExt, which has a chemical profile similar to the forms with ethnopharmacological use.

## Conclusions

ASE and chemometric calculations allowed us to obtain a standardized extract of gnaphaliin and helipyrone from *A*. *alata*. This extract has bacteriostatic properties and stimulates reactive oxygen species/nitric oxide, which is important to microbial and immunological responses. Relative to other extractive methodologies, such as infusion, the developed method was exhaustive and increased the concentrations for the compounds of interest more than eleven times. Furthermore, the chemical profiles of the different plant parts have similar compositions and differ from the *A*. *satureioides* substitute. This extract was not cytotoxic and has potential for use in oral hygiene product development, but requires pre-formulation studies.

## Supporting Information

S1 File ^1^H, ^13^C and Dept 135° NMR spectra of Gnaphaliin, Lepidissipyrone, Obtusifolin and Helipyrone. (DOCX)

S1 Table Factorial design and results. (DOCX)

S2 Table Response surface methodology and results. (DOCX)

S3 Table Antimicrobial and antioxidant activity of *Achyrocline alata* extracts. (DOCX)
