# Supplementary material for: Optimization and Technological Development Strategies of an Antimicrobial Extract from Achyrocline alata Assisted by Statistical Design
Source: PLoS One. 2015 Feb 24;10(2):e0118574. doi: 10.1371/journal.pone.0118574 (PMC4339785; doi:10.1371/journal.pone.0118574)
Supplement: S1 File — (DOCX) [file pone.0118574.s001.docx]

**File S1:** ^1^H, ^13^C and Dept 135º NMR spectra of Gnaphaliin, Lepidissipyrone, Obtusifolin and Helipyrone.

Chemical structure of Gnaphaliin.

Gnaphaliin NMR data (CDCl_3_; 300 MHz)

|  | **δ^1^H** | **δ^13^C** | **DEPT** |
| --- | --- | --- | --- |
| **2** | -- | 155.24 |  |
| **3** | -- | 139.66 |  |
| **4** | -- | 179.06 |  |
| **5** | -- | 157.54 |  |
| **6** | 6.42 (s. 1H) | 98.57 | CH |
| **7** | -- | 155.52 |  |
| **8** | -- | 126.77 |  |
| **9** | -- | 148.05 |  |
| **10** | -- | 105.75 |  |
| **1’** | -- | 130.46 |  |
| **2’** | 8.07 (dd, 2H) | 128.27 | CH |
| **3’** | 7.5 (m, 3H) | 128.77 | CH |
| **4’** | 7.5 (m, 3H) | 131.07 | CH |
| **5’** | 7.5 (m, 3H) | 128.77 | CH |
| **6’** | 8.10 (dd, 2H) | 128.27 | CH |
| **11 (OCH_3_)** | 3.84 (s, 3H) | 60.41 | CH_3_ |
| **12 (OH)** | -- | -- |  |
| **13 (OH)** | 12.34 (s, 1H) | -- |  |
| **14 (OCH_3_)** | 3.97 (s, 3H) | 61.94 | CH_3_ |


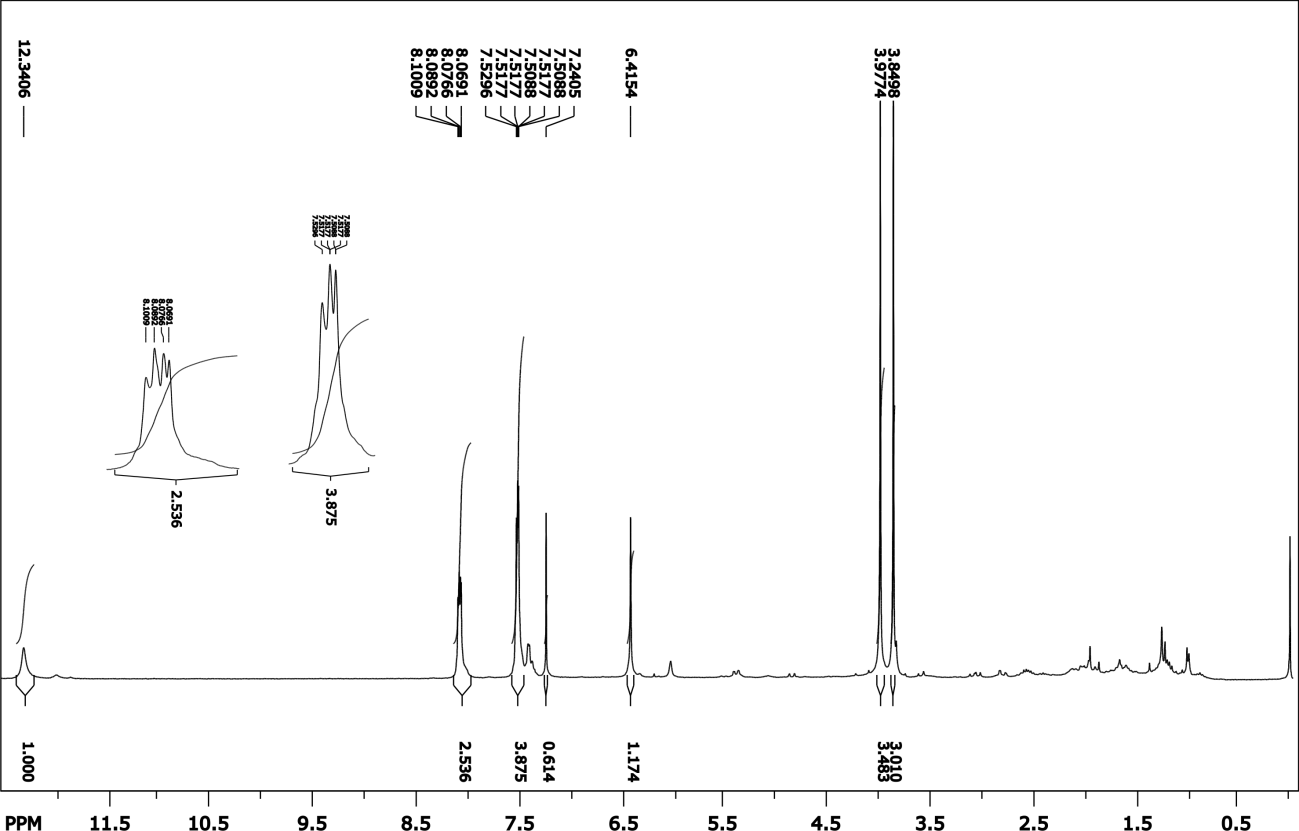

^1^H NMR spectrum of gnaphaliin (CDCl_3_; 300 MHz)

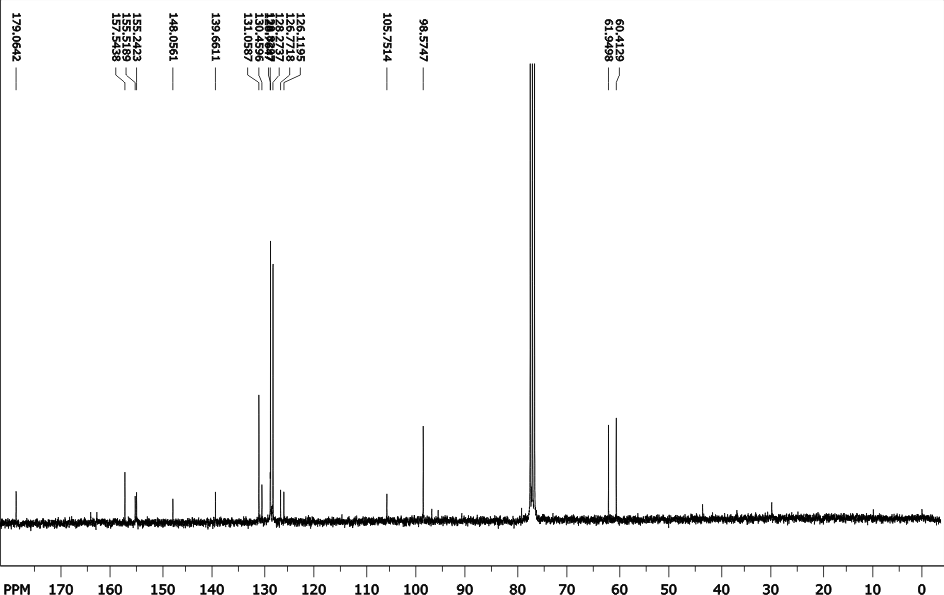


^13^C NMR spectrum of gnaphaliin (CDCl_3_; 300 MHz)


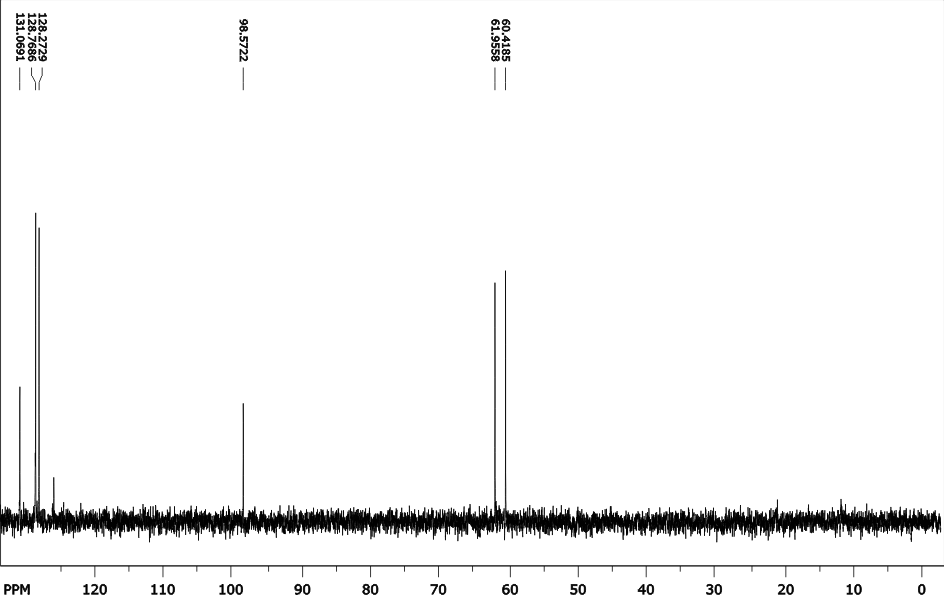

DEPT 135º NMR spectrum of gnaphaliin (CDCl_3_; 300 MHz)

**Chemical structures of Lepidissipyrone (Left) and Obtusifolin (Right).**

**Lepidissipyrone and Obtusifolin NMR data.**

|  | **FXE2** | | | **FXE4** | | |
| --- | --- | --- | --- | --- | --- | --- |
|  | **δ^1^H** | **δ^13^C** | **DEPT** | **δ^1^H** | **δ^13^C** | **DEPT** |
| **2** | 5.53 (dd, 1H, 2,5Hz/13,2Hz) | 81.5 | CH | 5.37 (dd, 1H, 3.1Hz/12,6Hz) | 79.1 | CH |
| **3a** | 2.83 (dd, 1H, 2,5Hz, 17,3Hz) | 42.7 | CH_2_ | 2.82 (dd, 1H, 3,1Hz, 17,2Hz) | 42.9 | CH_2_ |
| **3b** | 3.26 (dd, 1H, 13,2Hz, 17.3Hz) |  |  | 3.06 (dd, 1H, 12,6Hz, 17,2Hz) |  |  |
| **4** | -- | 194.7 | -- | -- | 196.2 | -- |
| **5** | -- | 157.7 | -- | -- | 158.9 | -- |
| **6** | -- | 104.9 | -- | 6.16 (s, 1H) | 98.2 | CH |
| **7** | -- | 163.1 | -- | -- | 161.6 | -- |
| **8** | 6.20 (s, 1H) | 99.6 | CH | -- | 106.7 | -- |
| **9** | -- | 165.8 | -- | -- | 166.0 | -- |
| **10** | -- | 102.3 | -- | -- | 102.1 | -- |
| **11** | 3.57 (s, 2H) | 17.4 | CH_2_ | 3.62 (s, 2H) | 19.1 | CH_2_ |
| **12** | -- | 101.1 | -- | -- | 101.6 | -- |
| **13** | -- | 166.8 | -- | -- | 169.1 | -- |
| **14** | -- | 107.6 | -- | -- | 108.0 | -- |
| **15** | -- | 161.8 | -- | -- | 161.3 | -- |
| **16** | 2.53 (q, 2H) | 24.3 | CH_2_ | 2.53 (q, 2H) | 24.3 | CH_2_ |
| **17** | 1.18 (t, 3H) | 11.5 | CH_3_ | 1.17 (t, 3H) | 11.6 | CH_3_ |
| **18** | 1.86 (s, 3H) | 9.2 | CH_3_ | 1.86 (s, 3H) | 9.4 | CH_3_ |
| **19** | -- | 168.9 | -- | -- | 169.6 | -- |
| **1’** | -- | 136.5 | -- | -- | 138.2 | -- |
| **2’ e 6’** | 7.53 (m, 5H) | 126.9 | CH | 7.40 (m, 5H) | 126.0 | CH |
| **3’ e 5’** |  | 129.3 | CH |  | 128.8 | CH |
| **4’** |  | 130.0 | CH |  | 128.8 | CH |


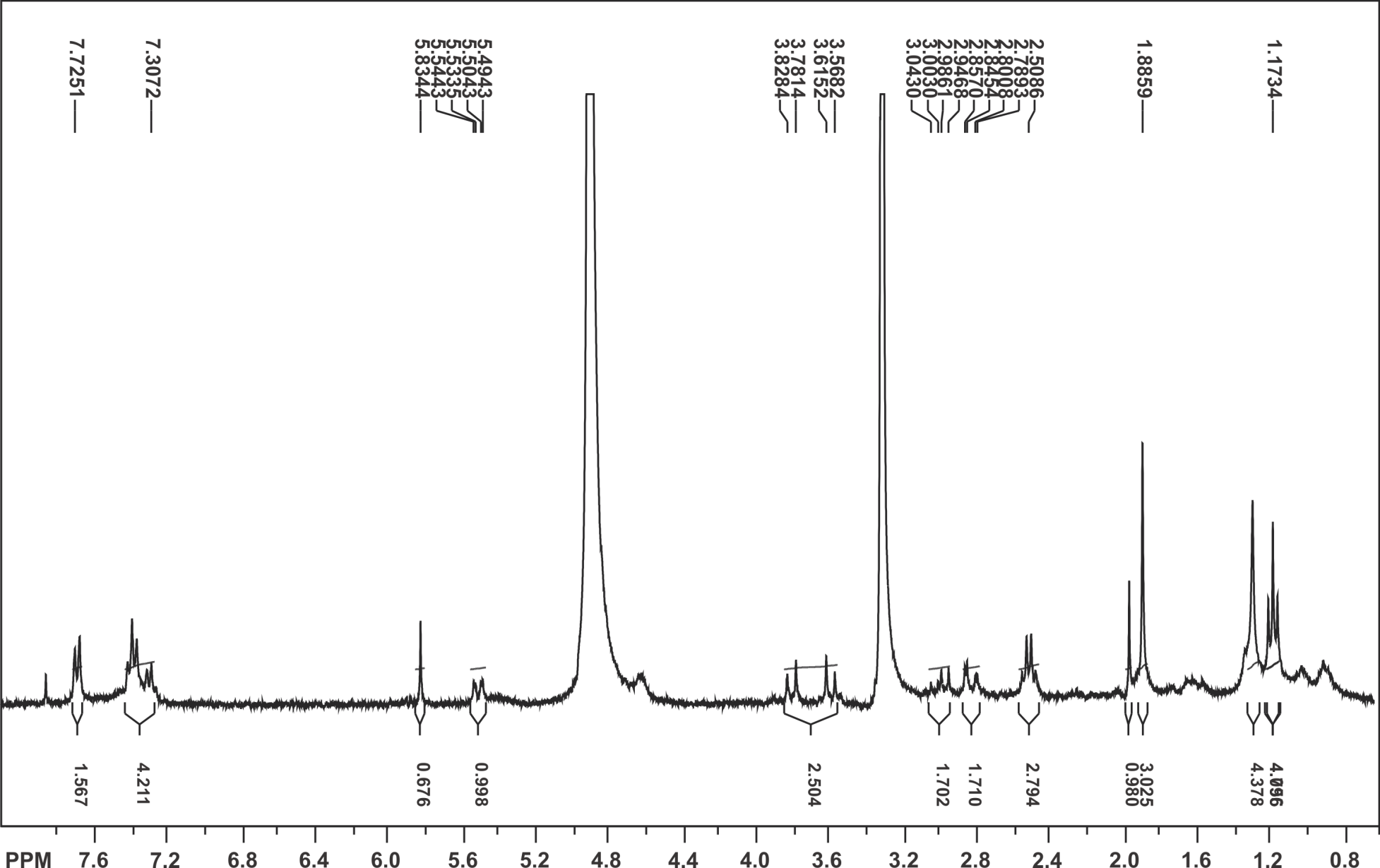

**^1^H NMR espectrum of Lepidissipyrone (MeOD; 300 MHz)**

**
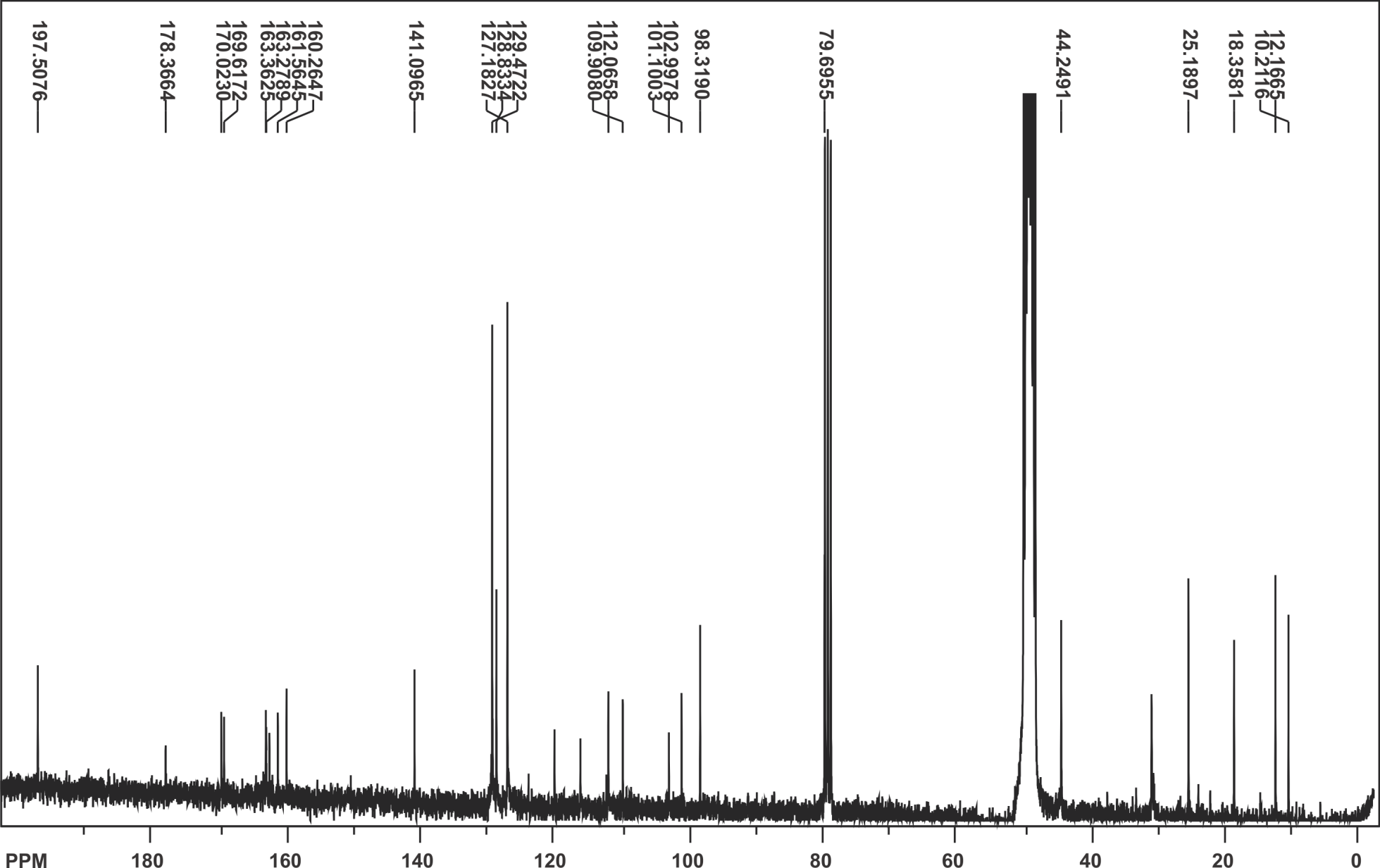
**

**^13^C NMR espectrum of Lepidissipyrone (MeOD; 300 MHz)**

**
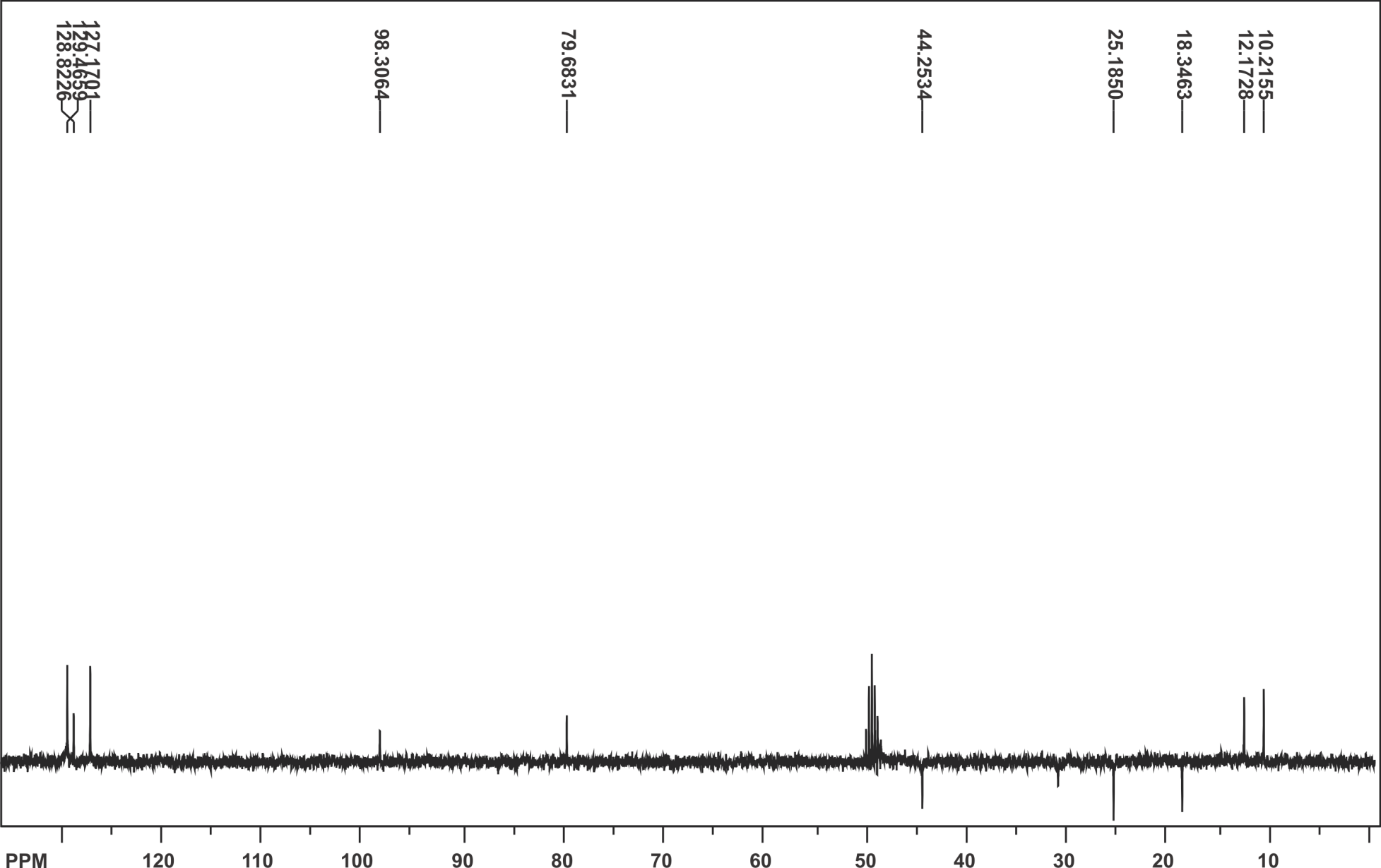
**

**DEPT 135º NMR espectrum of Lepidissipyrone (MeOD; 300 MHz)**

**
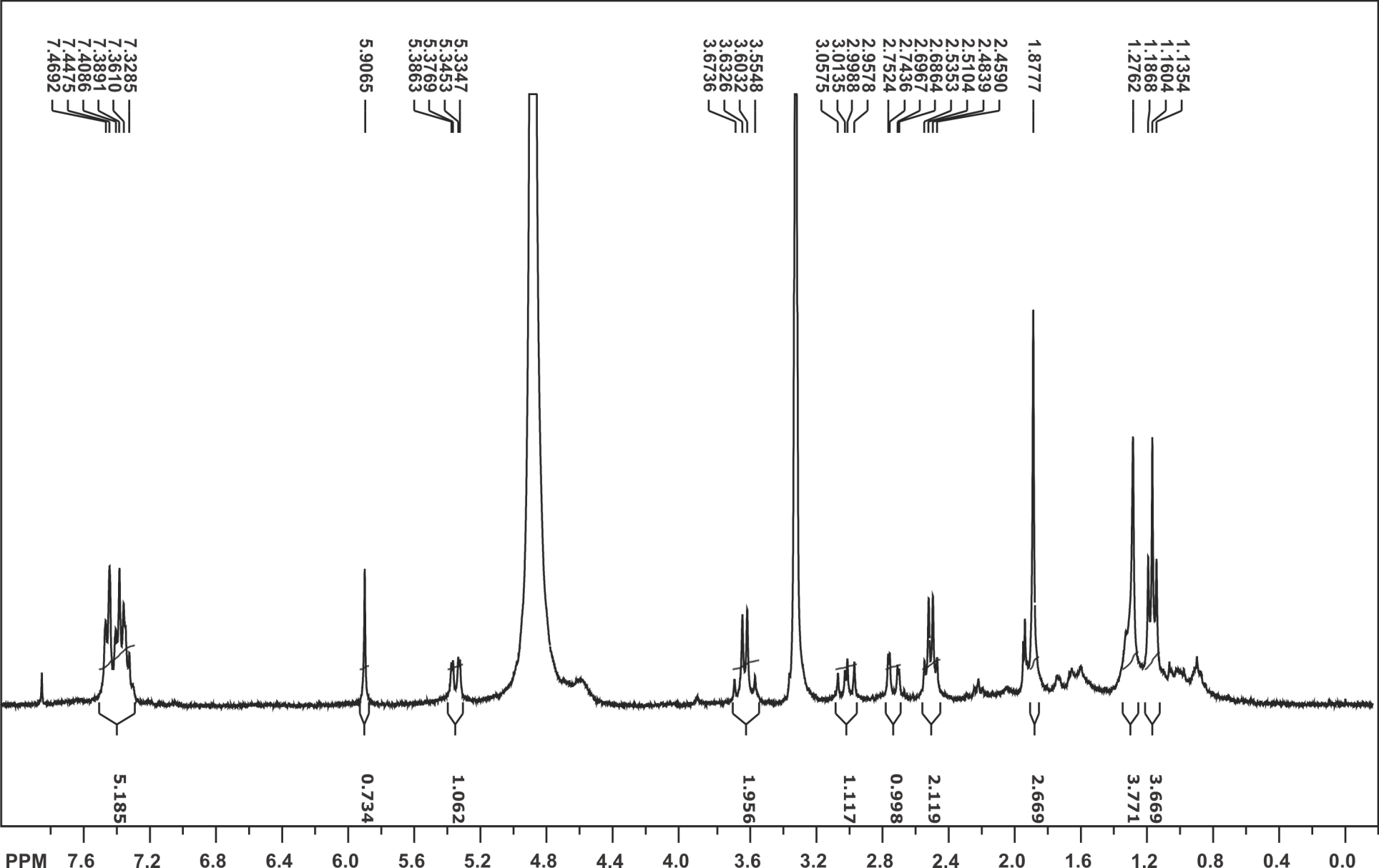
**

**^1^H NMR espectrum of Obtusifolin (MeOD; 300 MHz)**

**
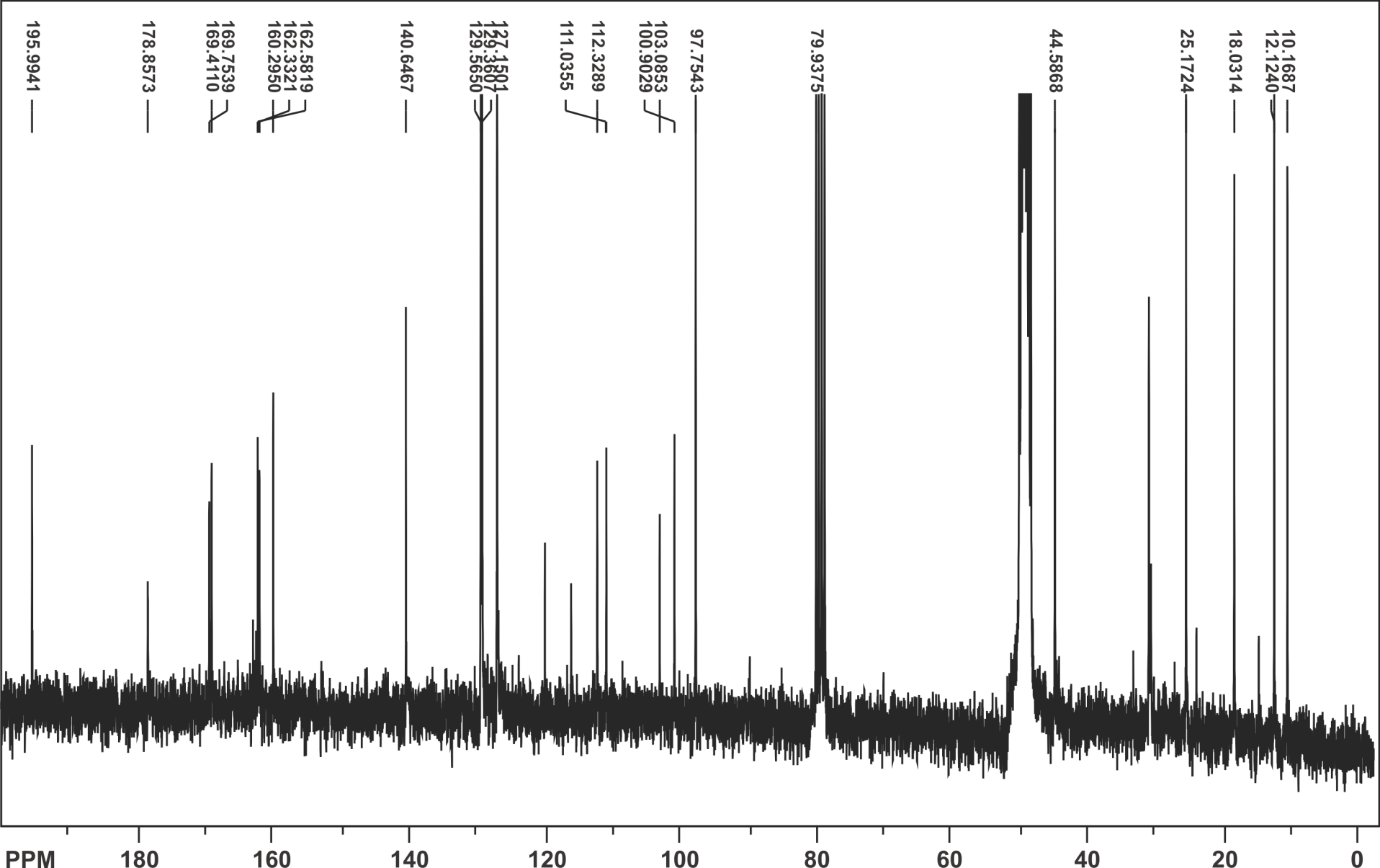
**

**^13^C NMR espectrum of Obtusifolin (MeOD; 300 MHz)**

**
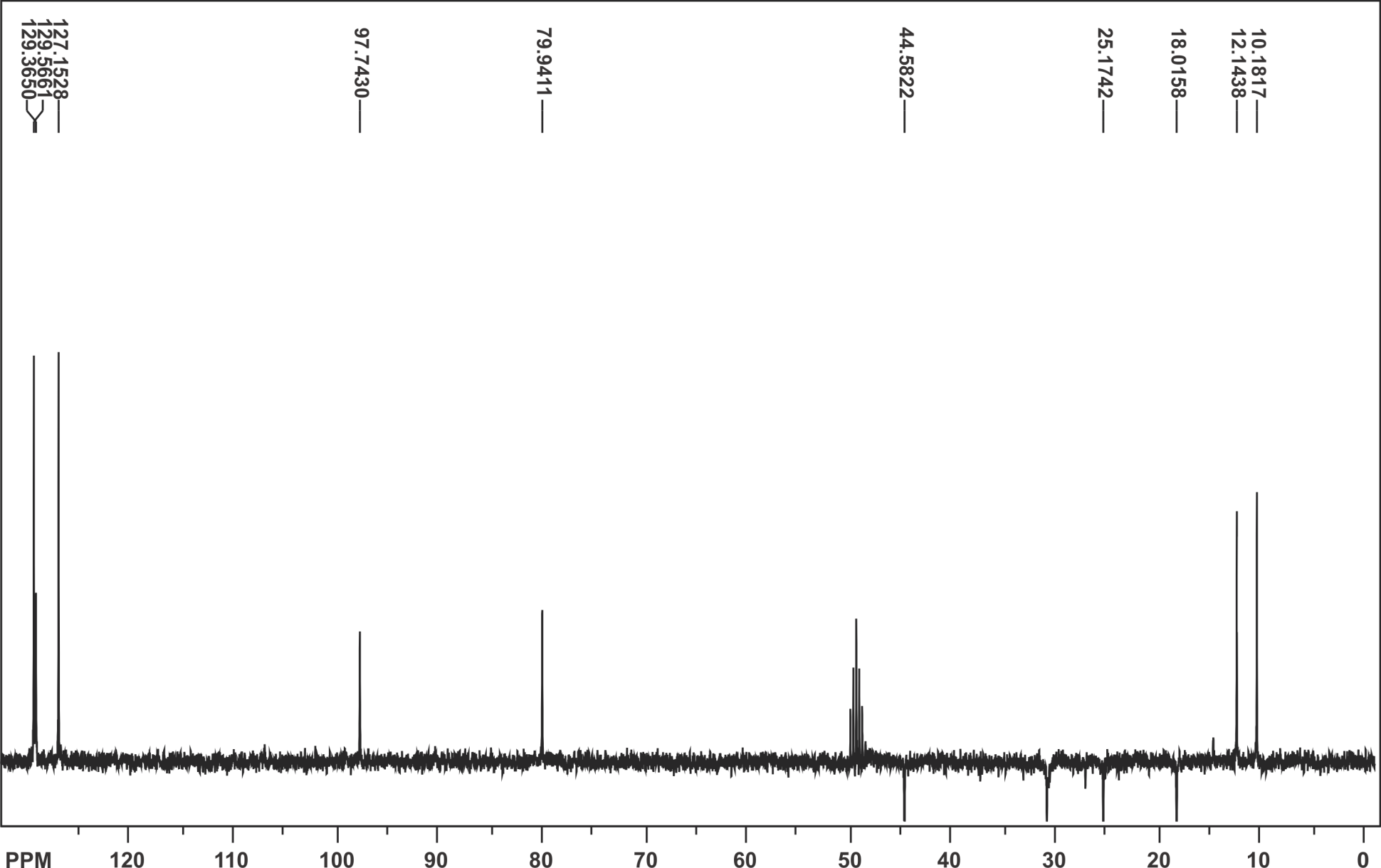
**

**DEPT 135º NMR espectrum of Obtusifolin (MeOD; 300 MHz)**

Helipyrone chemical structure.

Helipyrone NMR data.

|  | **δ^1^H** | **δ^13^C** | **DEPT** |
| --- | --- | --- | --- |
| 1 | 3.54 (s,2H) | 19.1 | CH_2_ |
| 2 | -- | 101.68 | -- |
| 3 | -- | 168.73 | -- |
| 4 | -- | 108.82 | -- |
| 5 | -- | 161.35 | -- |
| 6 | 2.56 (q, 4H) | 24.3 | CH_2_ |
| 7 | 1.20 (t, 6H) | 11.58 | CH_3_ |
| 8 | 1.96 (s, 6H) | 9.38 | CH_3_ |
| 9 | -- | 169.56 | -- |

CDCl_3_, 300 MHz


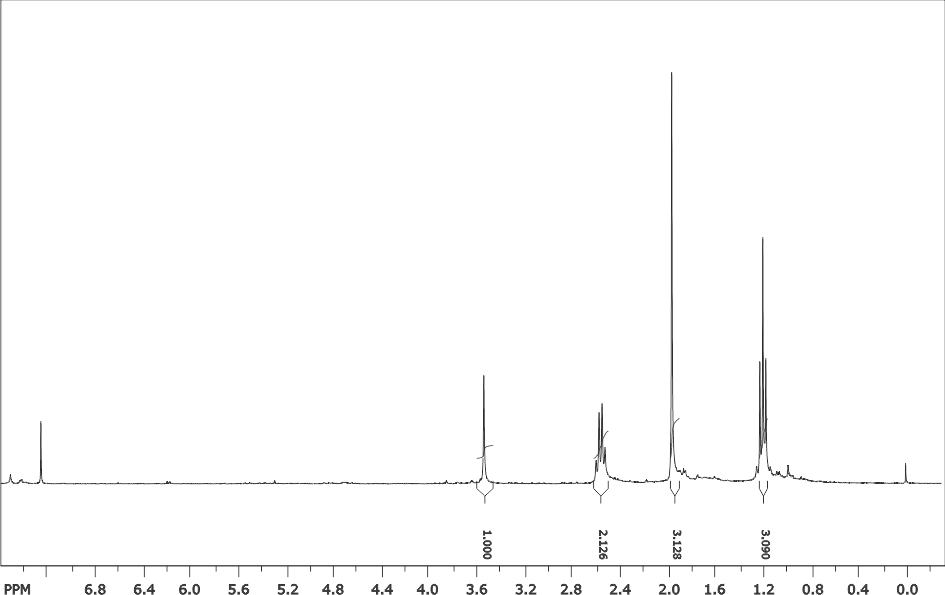

**^1^H NMR espectrum of Helipyrone (CDCl_3_; 300 MHz)**


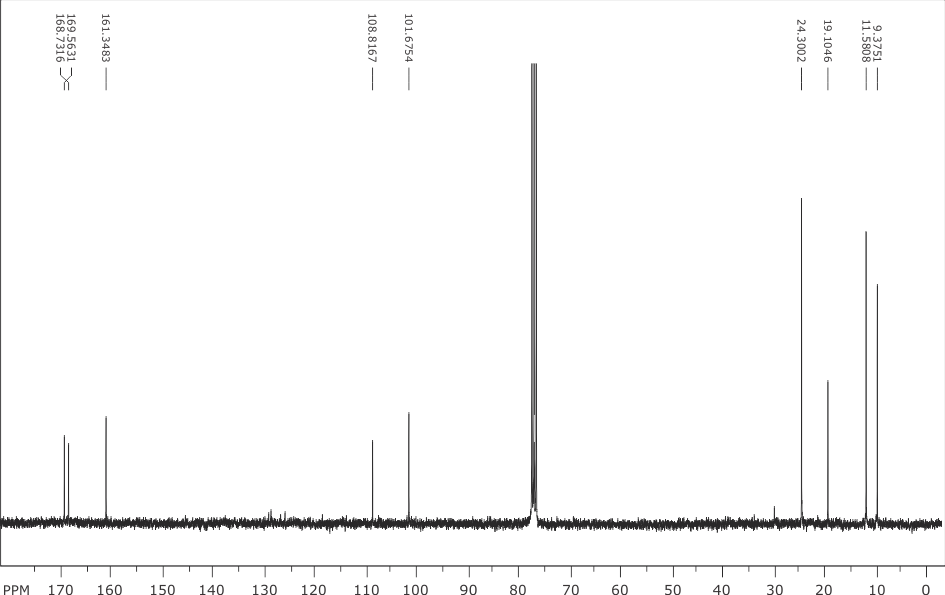

**^13^C NMR espectrum of Helipyrone (CDCl_3_; 300 MHz)**


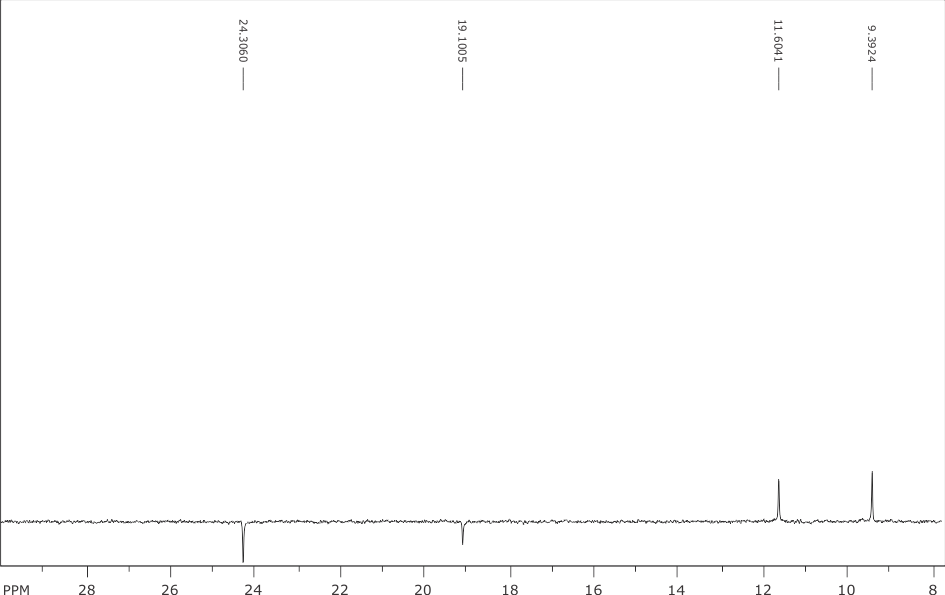

**DEPT 135º NMR espectrum of Helipyrone (CDCl_3_; 300 MHz)**
