# Supplementary material for: Optimization and Technological Development Strategies of an Antimicrobial Extract from Achyrocline alata Assisted by Statistical Design
Source: PLoS One. 2015 Feb 24;10(2):e0118574. doi: 10.1371/journal.pone.0118574 (PMC4339785; doi:10.1371/journal.pone.0118574)
Supplement: S1 Table — (DOCX) [file pone.0118574.s002.docx]

Table S1: Factorial design and results.

| **Run Order** | **HX/Ace** | **T (°C)** | **Cycles** | **t (min)** | **Ethanol** | **Yield (mg)** | **% Polic.** | **% Inat. C.** |
| --- | --- | --- | --- | --- | --- | --- | --- | --- |
| 1 | 75/25 | 100 | 3 | 3 | 70 | 14 | 9.98 | 59.32 |
| 2 | 100/0 | 70 | 5 | 1 | 90 | 4.7 | 20.36 | 14.08 |
| 3 | 100/0 | 70 | 1 | 1 | 50 | 2.5 | 18.83 | 7.17 |
| 4 | 100/0 | 130 | 1 | 5 | 50 | 2.6 | 22.59 | 9.39 |
| 5 | 100/0 | 130 | 1 | 5 | 50 | 1.4 | 21.65 | 14.76 |
| 6 | 50/50 | 130 | 1 | 1 | 90 | 9.2 | 10.09 | 57.94 |
| 7 | 100/0 | 70 | 5 | 1 | 90 | 4.7 | 20.56 | 17 |
| 8 | 100/0 | 130 | 5 | 5 | 90 | 6.6 | 18.73 | 24.45 |
| 9 | 50/50 | 130 | 5 | 1 | 50 | 15 | 6.85 | 70.95 |
| 10 | 50/50 | 70 | 5 | 5 | 50 | 1.4 | 9.18 | 62.64 |
| 11 | 50/50 | 130 | 5 | 1 | 50 | 9 | 8.56 | 65.84 |
| 12 | 50/50 | 70 | 5 | 5 | 50 | 1 | 9.8 | 62.02 |
| 13 | 100/0 | 70 | 1 | 1 | 50 | 2.6 | 23.34 | 6.49 |
| 14 | 50/50 | 70 | 1 | 5 | 90 | 7.7 | 9.09 | 68.19 |
| 15 | 50/50 | 70 | 1 | 5 | 90 | 13 | 9.97 | 51.65 |
| 16 | 50/50 | 130 | 1 | 1 | 90 | 19.8 | 9.3 | 51.95 |
| 17 | 100/0 | 130 | 5 | 5 | 90 | 6.6 | 19.09 | 19.3 |

**LEGEND:** **Run Order:** order of extractions; **HX/Ace:** hexane/acetone percentage used for extraction; **T (°C):** temperature; **Cycles:** cycles of extraction; **t (min):** extraction time of each cycle in minutes; **Ethanol:** ethanol/water percentage used to partition; **Yield (mg):** yield in milligrams; **% Polic:** active polyketides percentage obtained according to HPLC analysis; **% Inat C:** inactive compounds percentage obtained by HPLC analysis.
