# Supplementary material for: Optimization and Technological Development Strategies of an Antimicrobial Extract from Achyrocline alata Assisted by Statistical Design
Source: PLoS One. 2015 Feb 24;10(2):e0118574. doi: 10.1371/journal.pone.0118574 (PMC4339785; doi:10.1371/journal.pone.0118574)
Supplement: S2 Table — (DOCX) [file pone.0118574.s003.docx]

Table S2: Response surface methodology and results.

| **Run Order** | **T** | **Hx/**  **EtAce** | **Rendimento (mg)** | **Gnap % max** | **Helip % max** | **Gnap%+Helip%**  **100** |
| --- | --- | --- | --- | --- | --- | --- |
| 1 | 180 | 80 | 44.5 | 100 | 70.63 | 1.70 |
| 2 | 140 | 80 | 25 | 31.93 | 12.39 | 0.44 |
| 3 | 140 | 90 | 23.6 | 64.80 | 63.27 | 1.28 |
| 4 | 180 | 100 | 14.9 | 50.79 | 52.89 | 1.03 |
| 5 | 140 | 90 | 28.1 | 59.29 | 66.26 | 1.25 |
| 6 | 140 | 100 | 14.9 | 71.12 | 91.53 | 1.62 |
| 7 | 180 | 90 | 23.1 | 91.04 | 81.25 | 1.72 |
| 8 | 100 | 80 | 7 | 31.34 | 16.08 | 0.47 |
| 9 | 140 | 90 | 23.9 | 72.90 | 100 | 1.72 |
| 10 | 100 | 100 | 13.9 | 45.04 | 62.22 | 1.07 |
| 11 | 100 | 90 | 13.7 | 26.43 | 29.70 | 0.56 |
| 12 | 140 | 90 | 31.9 | 65.16 | 71.10 | 1.36 |
| 13 | 140 | 90 | 25 | 69.05 | 72.38 | 1.41 |

**LEGEND:** **Run Order:** order of extractions; **T (°C):** temperature; **HX/ETAce:** hexane/ethyl acetate percentage used for extraction; **Yield (mg):** yield in milligrams; **Gnap % max:** maximum gnaphaliin yield; **Helip % max:** maximum helipyrone yield; **Gnap%+Helip%/100:** maximum helipyrone adedd gnaphallin yield.
