# Supplementary material for: Optimization and Technological Development Strategies of an Antimicrobial Extract from Achyrocline alata Assisted by Statistical Design
Source: PLoS One. 2015 Feb 24;10(2):e0118574. doi: 10.1371/journal.pone.0118574 (PMC4339785; doi:10.1371/journal.pone.0118574)
Supplement: S3 Table — (DOCX) [file pone.0118574.s004.docx]

**Table S3: Antimicrobial and antioxidant activity of *Achyrocline alata* extracts.**

| Extract | MIC | Antioxidant activity (IC_50_) |
| --- | --- | --- |
| EExt | 1000 μg/mL | 19.8 |
| HExt | 500 μg/mL | ---- |
| HExt-EP | 62.5 μg/mL | 609 |

* Antioxidant standard quercetin (IC_50_ = 3.4).
